# A Study on the Gas/Humidity Sensitivity of the High-Frequency SAW CO Gas Sensor Based on Noble-Metal-Modified Metal Oxide Film

**DOI:** 10.3390/s23052487

**Published:** 2023-02-23

**Authors:** Haiyang Yang, Bin Shen, Xinlei Liu, Chunbo Jin, Tianshun Zhou

**Affiliations:** School of Safety Engineering, Heilongjiang University of Science and Technology, Harbin 150022, China

**Keywords:** SAW, Pd–Pt/SnO_2_/Al_2_O_3_ thin film, CO gas, gas sensitivity, humidity sensitivity

## Abstract

In order to improve the response characteristics of the surface acoustic wave (SAW) sensor to trace gases, a SAW CO gas sensor based on a Pd–Pt/SnO_2_/Al_2_O_3_ film with a high-frequency response performance is proposed in this paper. The gas sensitivity and humidity sensitivity of trace CO gas are tested and analyzed under normal temperatures and pressures. The research results show that, compared with the frequency response of the Pd–Pt/SnO_2_ film, the CO gas sensor based on a Pd–Pt/SnO_2_/Al_2_O_3_ film has a higher frequency response performance, and the sensor has high-frequency response characteristics to CO gas with a concentration in the range of 10–100 ppm. The average response recovery time of 90% ranges from 33.4 s to 37.2 s, respectively. When the CO gas with a concentration of 30 ppm is tested repeatedly, its frequency fluctuation is less than 5%, indicating that the sensor has good stability. In the range of relative humidity (RH) from 25% to 75%, it also has high-frequency response characteristics for CO gas with a 20 ppm concentration.

## 1. Introduction

As a large coal energy country, China’s coal production and coal storage levels are extremely high. However, there are many natural gas accidents caused by coal mining [1,2,3], with CH_4_ accidents accounting for the majority of these [4,5]. The latter refers to the accidents caused by trace toxic and harmful gases, such as CO, CO_2_, and H_2_S, as well as other trace gases [6,7]. The harmful impacts of such gases cannot be underestimated.

SAW sensors are gaining more and more attention as SAW technology is improving. Compared to other types of sensors, SAW sensors have greater advantages, such as high measurement accuracy, high sensitivity, small size, light weight, low power consumption, and the possibility of wireless passive [8,9,10]. Moreover, a wide range of gases can be detected by simply coating the sensitive area of the device with a sensitive material with different gas adsorption properties. These good features make the application of SAW sensors promising. However, there are also some challenges, as the response degree of different sensitive films to different gases is different, and the avenues of improving the response degree of SAW sensors to gases with greater efficiency and accuracy are currently subject to intense research activity among scholars.

In 1986, David et al. [11] used SAW gas sensors coated with different sensitive films to detect 11 chemical vapors. In 2003, Weimar et al. [12] used the thick film technique to develop a sensor with Pd material loaded on SnO_2_ to study the effect of water vapor on CO gas sensing. On the other hand, in 2009, Huang Guogang et al. [13] used the frequency division technique and achieved high-accuracy measurements of CO gas frequency by improving the test technique with the help of the frequency error multiplication method for acoustic surface wave sensors. In 2017, Wang Qingji et al. [14] prepared a Pd-doped SnO_2_ hollow structure using a hydrothermal synthesis method, and developed a CO sensor with high response values from this sensitive material. This sensor achieved a response value of 14.7 for 100 ppm CO. In 2019, Pengjian Wang [15] et al. fabricated a CO gas sensor based on a PdO-loaded SnO_2_ film, and the results showed that the sensor had the best sensitivity for 100 ppm CO at a specific temperature. In 2020, Xiaofeng Xu et al. [16] used the sol–gel method to prepare the SAW sensor with the ZnO–Al_2_O_3_ thin film, which can detect H_2_S gas with a gas-moisture sensitivity at room temperature, making significant progress in regards to the effect of humidity on gas. In 2022, Yuan Tongwei et al. [17] prepared a new type of CO gas sensing material with porous n–ZnO/p–Co_3_O_4_ nano sheets using a new method of etching forging. The sensors under this sensitive material have high response characteristics to CO gas and an excellent anti-interference ability.

Although significant research results have been achieved in the detection of trace toxic and hazardous gases, the response values for trace gases need to be higher and more accurate. Therefore, this chapter investigates ways to improve the frequency response of SAW sensors to gases. In this study, a high-frequency SAW CO gas sensor based on a Pd–Pt/SnO_2_/Al_2_O_3_ sensitive film was prepared, which has high response characteristics for the trace amounts of low-concentration CO gas generated in coal mines. It is also capable of detecting high frequencies for trace ppm levels of CO gas at room temperature, and has a high-frequency response performance at different RH environments.

## 2. Experimental Part

### 2.1. Sensing Mechanism

#### 2.1.1. Elastic Loading Effect

The elastic load means that a specific gas is adsorbed on the surface of the SAW sensor, and that the elastic modulus before and after the sensitive film will change, resulting in propagation speed changes, which will affect the center frequency of the device. The relationship between the frequency offset and elastic modulus of the SAW sensor is shown in Formula (1) [18].

(1) $$\Delta f=c_{e}f_{0}{}^{2}h\left[\left(\frac{4u}{v_{0}^{2}}\right)\times \left(\frac{u+\lambda }{u+2\lambda }\right)\right]$$

where $c_{e}$ is the elastic sensitivity coefficient, $f_{0}$ is the center frequency of the SAW sensor, h is the thickness of the sensitive film, $v_{0}$ is the initial wave velocity of the SAW sensor, u is the volume component of the elastic modulus, and λ is the shear modulus of the elastic modulus.

When the elastic load changes, the center frequency of the device also changes. When the value of the bulk component of the elastic modulus is less than 10^6^ Pa, the effect of the elastic load on the device is almost zero, and when the value of the shear modulus of the elastic modulus is greater than 10^9^ Pa, the elastic load is generated at this time. The effect cannot be ignored, and it will cause a large shift in the center frequency of the device.

#### 2.1.2. Conductivity Loading Effect

The relationship between the shift of the SAW sensor frequency response and the conductivity is shown in Formula (2).

(2) $$\Delta f=-\frac{K^{2}}{2}f_{0}\left(\frac{\sigma_{s}^{2}}{\sigma_{s}^{2}+v_{0}^{2}C_{s}}\right)$$

where $\sigma_{s}$ is the conductivity on the sensitive film, $K^{2}$ is the electromechanical coupling coefficient of the piezoelectric substrate, and $C_{s}$ is the dielectric constant.

After the gas is adsorbed by the sensitive membrane, its conductivity and dielectric constant will change, resulting in a large frequency shift. However, the greater the conductivity, the better. Only when the conductivity is in a proper range can the SAW gas sensor have the best sensitivity.

#### 2.1.3. Mass Loading Effect

The relationship between the frequency offset of the sensor and the quality change in its sensitive film is shown in Formula (3) [19].

(3) $$\Delta f=f_{0}{}^{2}\left(k_{1}+k_{2}\right)\Delta m$$

where Δf is the frequency offset of the sensor, $f_{0}$ is the center frequency of the sensor, $k_{1}\;\mathrm{and}\;k_{2}$ is the constant of the piezoelectric substrate material, and Δm is the mass change of the sensitive film.

The constants $k_{1}$ and $k_{2}$ are obtained differently with different piezoelectric substrate materials. When the sensitive film makes contact with a specific gas, the gas molecules will combine with the sensitive film and immerse in it, thus increasing the quality of the film. Before and after the adsorption of gas on the sensitive film, the quality of the sensitive film will change, which will affect the center frequency of the sensor, resulting in a frequency offset.

### 2.2. Preparation and Characterization of Sensitive Films

In this paper, metal PdCl_2_ solutions and PtCl_2_ solutions (Pd, Pt content ≥ 59%, Shanghai Jiuling Chemical Co., Shanghai, China), SnO_2_ powder (around 45 nm, 99.99% purity, Shanghai Jiuling Chemical Co., Shanghai, China), and Al_2_O_3_ powder (20–50 nm, 99.99% purity, Shanghai Jiuling Chemical Co., Shanghai, China) were used as basic raw materials. First, SnO_2_ powder (13.2 g) and Al_2_O_3_ powder (3 g) were taken in the molar mass ratio of Sn:Al = 1:1, and then the SnO_2_ powder was mixed with an appropriate amount of deionized water and stirred thoroughly, Afterwards, the Al_2_O_3_ powder was added and then stirred at a constant speed at 30 °C for 2 h to allow the Al_2_O_3_ material to be evenly adhered to. The SnO_2_/Al_2_O_3_ mixture was then obtained by stirring for 2 h at a constant speed at 30 °C, allowing the Al_2_O_3_ material to adhere uniformly to the SnO_2_ material. Subsequently, about 0.1 mL of PdCl_2_ solution and PtCl_2_ solution was added to the SnO_2_/Al_2_O_3_ mixture via a micro syringe, the Pd–Pt/SnO_2_/Al_2_O_3_ films were obtained by standing for 5 h, evaporating in a water bath at 60 °C, before being heated for 2 h in muffle furnace at 500 °C (Model: TX–08, Shanghai KEA Experimental Instrument Factory, Shanghai, China). It was then removed, left to cool, and grinded for use.

**Figure 1:**
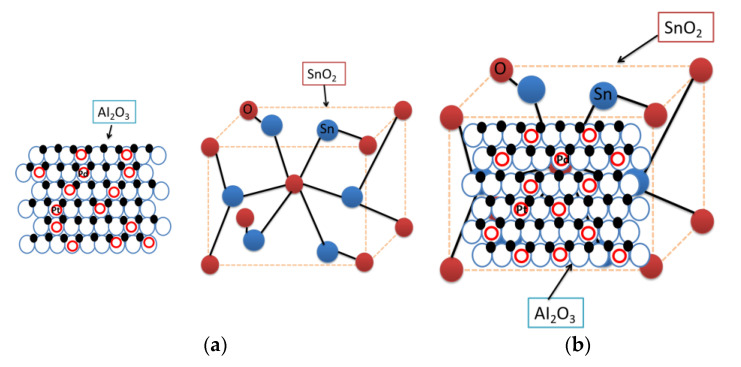
Schematic diagram of the material structure: (**a**) SnO_2_ and Al_2_O_3_ structure; and (**b**) SnO_2_/Al_2_O_3_ composite structure.

The reason for first adding SnO_2_ powder and deionized water, followed by Al_2_O_3_ powder, is because SnO_2_ materials generally exhibit three crystal structures (tetragonal, orthorhombic, and cubic), the former of which indicates the most common crystal structure for SnO_2_, while Al_2_O_3_ has a mesoporous structure with a high specific surface area [20] (≥230 m^2^/g) and can better adhere to the surface of SnO_2_. This increases the active sites for gas adsorption and improves its sensitivity. The noble metal has good catalytic and low-temperature activity and is attached to the surface of the material to shorten the response recovery time. A diagram of the lamination of the film material is shown in Figure 1 (with the frontal lamination effect as an example).

**Figure 2:**
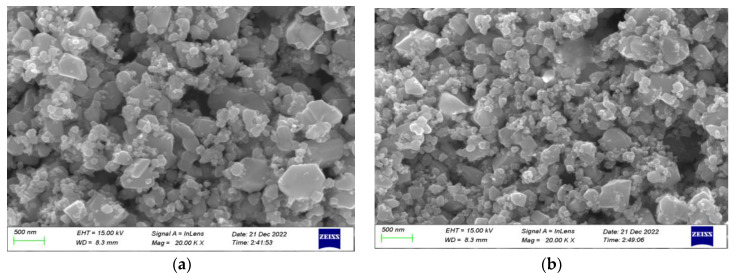
SEM characterization of sensitive films: (**a**) the Pd–Pt/SnO_2_ thin film; and (**b**) the Pd–Pt/SnO_2_/Al_2_O_3_ thin film.

Figure 2 shows the morphological results of the Pd–Pt/SnO_2_ thin films and Pd–Pt/SnO_2_/Al_2_O_3_ thin films. As the figure shows, the microscopic dimensions of the Pd–Pt/SnO_2_ material were 60–80 nm and the perimetric dimensions of the Pd–Pt/SnO_2_/Al_2_O_3_ material were 20–60 nm. The sensitive film had an increased specific surface area and more active sites after adding Al_2_O_3_, and its adsorption effect on gases was enhanced.

**Figure 3:**
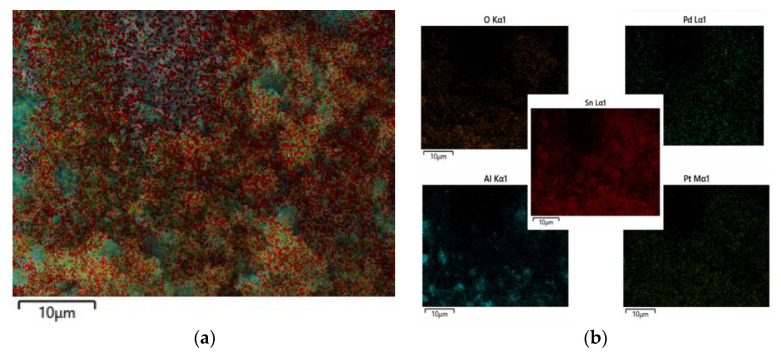
Elemental distribution of Pd–Pt/SnO_2_/Al_2_O_3_ films: (**a**) elegional elemental distribution maps; and (**b**) single elemental distribution maps.

**Figure 4:**
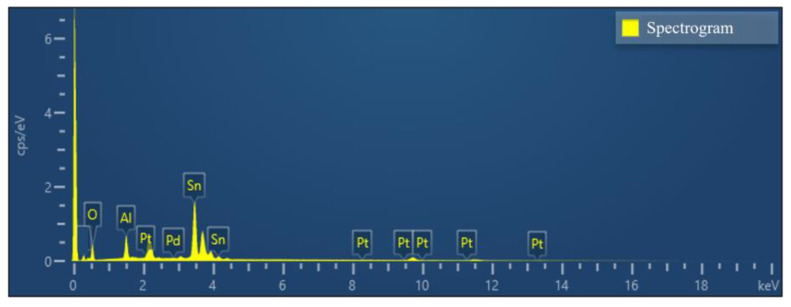
Total spectrum of Pd–Pt/SnO_2_/Al_2_O_3_ thin films with elemental distribution.

Figure 3 and Figure 4 and Table 1 shows the results of the energy spectrum characterization analysis of the Pd–Pt/SnO_2_/Al_2_O_3_ thin film. Figure 3 shows that each element (Al, Sn, O, Pd, and Pt) was present in the composite film and can be uniformly distributed, proving that the composite film prepared was consistent with the above theoretical analysis.

## 3. Results

### 3.1. Construction of SAW Gas Sensor Performance Test System

**Figure 5:**
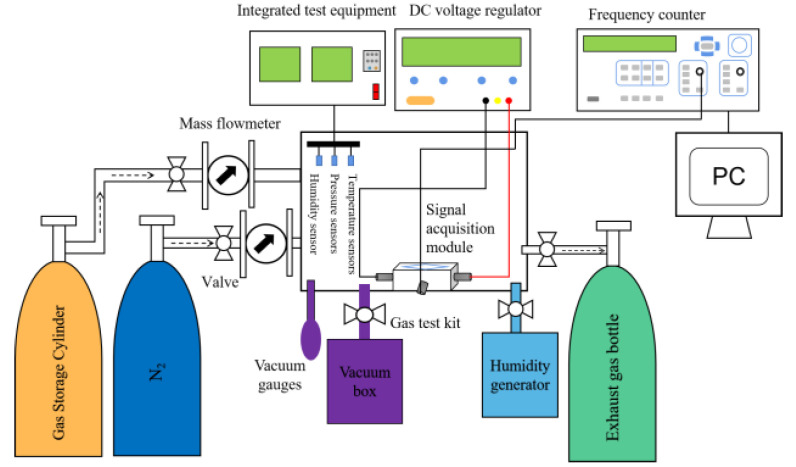
Schematic diagram of the gas susceptibility test system.

The main purpose of building the test system of the SAW CO gas sensor was to separate the interference factors such as the temperature, humidity, and pressure from the test system, and discuss and analyze them separately, thereby reducing the test error. Information on the CO gas performance test system and the test apparatus is shown in Figure 5 and Table 2, respectively.

In this experiment, we only analyzed the gas sensitivity and humidity sensitivity of the gas. Therefore, pressure sensors and temperature sensors were needed to control the variables to ensure that the experiment was conducted at room temperature and room pressure, and real-time monitoring was carried out. Before testing the CO gas sensitivity, each piece of equipment was first debugged to check the air tightness of the whole test system, and then a vacuum pump was used to evacuate the vacuum in the test box for 30 min, followed by a vacuum gauge to observe the gas in the test box. During the test, N_2_ gas was introduced under a normal temperature, and the air pressure in the test box was always kept as a standard atmospheric pressure, so as to eliminate interference factors (such as the water vapor or impurity gas) that affect the sensitive membrane in the test box. The gas used in the test was a mixture of N_2_ as the reference gas and CO gas, and the flow rates of N_2_ and CO were then controlled by the mass flowmeter, so as to accurately control the CO concentration. When testing the humidity sensitivity of CO, the RH value of the test box was adjusted with the humidity generator. To test the gas humidity sensitivity of CO, the network analyzer was used to record the working frequency and other relevant parameters of the SAW sensor.

### 3.2. Sensitive Film RESPONSE Test 

**Figure 6:**
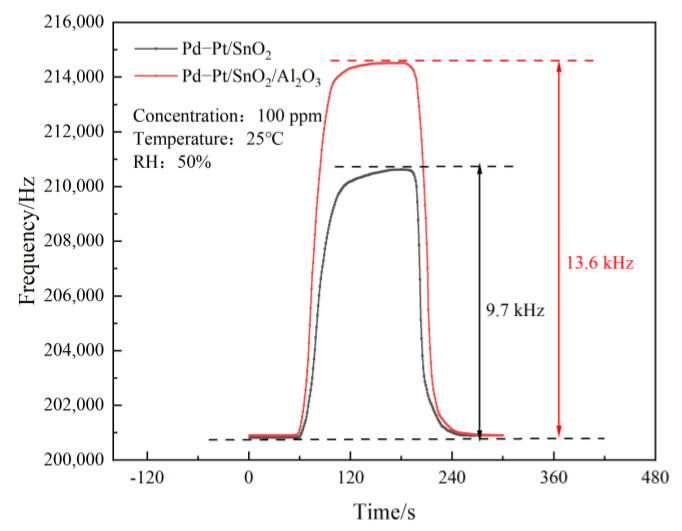
SAW sensor atrophicity test based on Pd–Pt/SnO_2_ and Pd–Pt/SnO_2_/Al_2_O_3_ thin films.

The CO concentration under the coal mine was not expected to exceed 24 ppm. Therefore, the test was conducted in a 10–100 ppm CO gas concentration. Based on the prepared Pd–Pt/SnO_2_ thin film and Pd–Pt/SnO_2_/Al_2_O_3_ thin film SAW sensors, the gas sensitivity of the 100 ppm CO gas was detected. When the working voltage was set to 12 V, the ambient temperature was controlled at room temperature (25 °C), the ambient RH was maintained at 50%, and the frequency response of the above two sensitive films to CO was tested. The results are shown in Figure 6.

The figure shows that the frequency response of the Pd–Pt/SnO_2_/Al_2_O_3_ film SAW sensor to the 100 ppm CO gas concentration (13.6 kHz) was significantly higher than that of the Pd–Pt/SnO_2_ film SAW sensor in the 100 ppm CO gas concentration (9.7 kHz). This shows that the Pd–Pt/SnO_2_/Al_2_O_3_ composite film can inherit the mesoporous structure of Al_2_O_3_, increases the specific surface area, and improve the sensitivity of the sensor.

### 3.3. Gas Sensitivity Test

**Figure 7:**
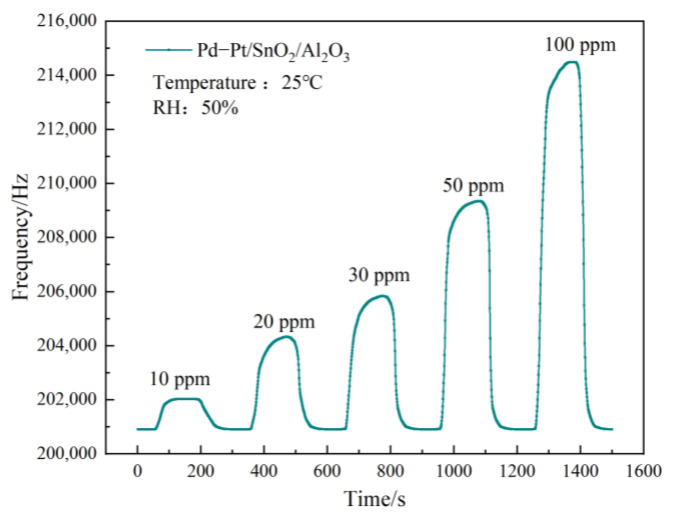
Dynamic response of different CO concentrations of SAW sensors.

Under the same test conditions, Figure 7 shows the SAW sensor based on the Pd–Pt/SnO_2_/Al_2_O_3_ sensitive film for the 10–100 ppm CO dynamic response of the gas concentration. The figure shows that the response frequency of the 10 ppm CO gas concentration was 1.1 kHz, and that the response frequency of the 100 ppm CO gas concentration reached 13.6 kHz, indicating that the SAW sensor under the membrane has high-frequency response characteristics to CO gas.

### 3.4. Response Recovery Characteristic Test

**Figure 8:**
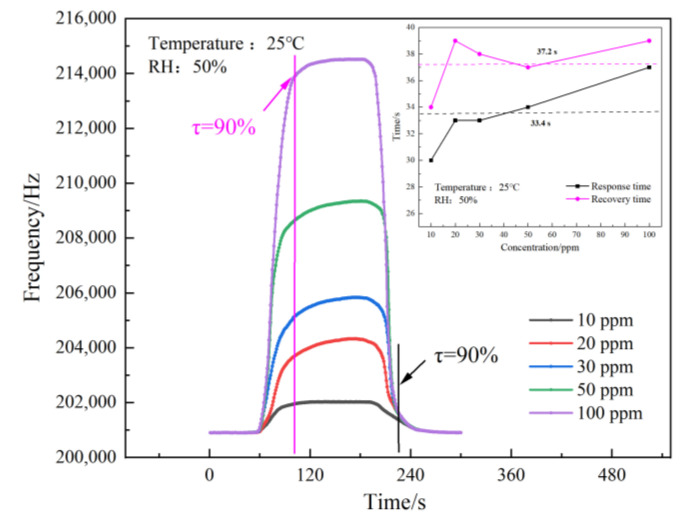
Response recovery time complex characteristic curve of the SAW sensor.

Under the same test conditions (25 °C, RH 50%), through the different CO gas concentrations configured, the SAW sensor of the Pd–Pt/SnO_2_/Al_2_O_3_ film was used to measure the 10 ppm to 100 ppm response recovery curve. The test results are shown in Figure 8. The figure shows that the SAW sensor based on the Pd–Pt/SnO_2_/Al_2_O_3_ composite film has a good response and recovery performance to different CO gas concentrations. Among them, 90% of the average response time equated to about 33.4 s, and 90% of the average recovery time equated to about 37.2 s. The response speed of the sensor was slightly faster than the recovery speed.

### 3.5. Stability Test

**Figure 9:**
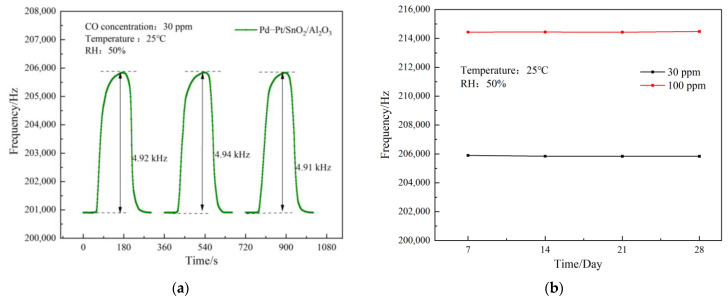
SAW sensor stability testing: (**a**) short-term stability; and (**b**) long-term stability.

The test conditions are the same as the above. The SAW sensor of the Pd–Pt/SnO_2_/Al_2_O_3_ sensitive film in the CO gas with a concentration of 30 ppm was used over four cycles to measure the stability of the sensor, as shown in Figure 9a. The amplitude changes in the frequency response of the composite membrane SAW sensor in each cycle were 4.92 kHz, 4.94 kHz, and 4.91 kHz, respectively. The test results show that the fluctuation in the frequency response was less than 5%. They also show that the sensor has good short-term stability.

Under the same test conditions, use the SAW sensor was used with the Pd–Pt/SnO_2_/Al_2_O_3_ composite film to test the CO gas within 30 days, and the data were recorded every 7 days to investigate the long-term stability of the SAW sensor. For this long-term stability test, CO gas with concentrations of 30 ppm and 100 ppm were selected as the two groups of experimental objects, and the test results are shown in Figure 9b. The figure shows that the sensor had a stable response to 30 ppm and 100 ppm CO gas within 30 days of testing, demonstrating that the SAW sensor based on the Pd–Pt/SnO_2_/Al_2_O_3_ composite sensitive film has good long-term stability for CO gas.

### 3.6. Humidity Test

**Figure 10:**
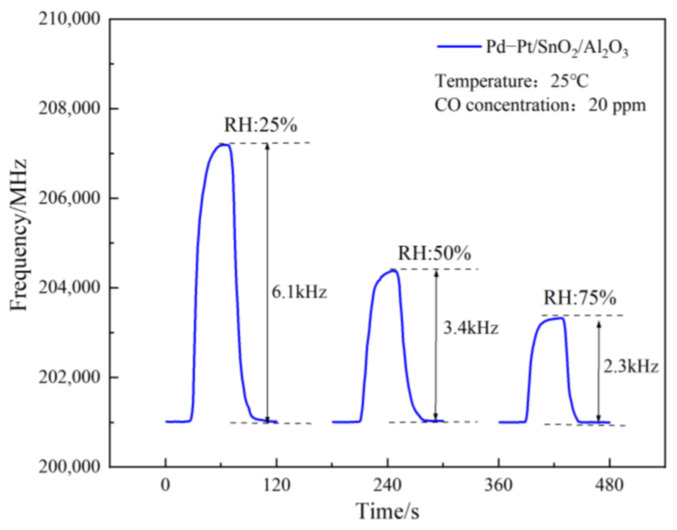
SAW sensor moisture sensitivity test.

The SAW gas sensor based on the Pd–Pt/SnO_2_/Al_2_O_3_ sensitive film uses CO gas with a concentration of 20 ppm, and conducts short-term humidity sensitivity performance tests of CO gas under three humidity conditions of 25%, 50%, and 75%, Three sets of experiments with different humidity levels were tested independently. The test results are shown in Figure 10 below.

The figure demonstrates that, with the increase in test humidity, the response frequency of the SAW gas sensor to CO decreased, the RH increased from 25% to 50% (and then to 75%), and the frequency decreased from 6.1 kHz to 3.4 kHz (and then to 2.3 kHz). The Pd–Pt/SnO_2_/Al_2_O_3_ sensitive film’s response frequency to CO gas decreased, possibly because the higher humidity meant more H_2_O molecules could be deposited on the film. On the one hand, H_2_O molecules occupied the active sites on the film by adsorbing CO gas. On the other hand, after more H_2_O molecules were adsorbed on the film, not only did the conductivity and elastic modulus change to some extent, but the quality of the film also improved. Under the mechanism of the mass loading effect, the response frequency of the film for detecting gas decreased. However, the SAW gas sensor based on the Pd–Pt/SnO_2_/Al_2_O_3_ sensitive film was still found to have a good humidity sensitive detection performance for CO gas in a certain humidity environment

## 4. Conclusions

In this paper, a high-frequency response SAW CO gas sensor with the Pd–Pt/SnO_2_/Al_2_O_3_ sensitive film was prepared. The gas sensitivity and humidity sensitivity of trace toxic CO gas were tested and analyzed at 25 °C and 50% RH. The following conclusions can be drawn from the test results. In the detection of 100 ppm CO gas concentration, the Pd–Pt/SnO_2_/Al_2_O_3_ sensitive film had a higher response frequency than the Pd–Pt/SnO_2_ sensitive film. In the range of 10–100 ppm CO gas concentration, the sensor based on the Pd–Pt/SnO_2_/Al_2_O_3_ sensitive film had high-frequency response characteristics, and the average response time and recovery time of 90% were 33.4 s and 37.2 s, respectively. When the CO gas with a 30 ppm concentration was tested repeatedly, its frequency response fluctuation was less than 5%, indicating that the sensor has good stability. In the range of RH of 25–75%, with the increase of RH, mainly due to the mass loading effect, the response frequency decreased, but it still had good response characteristics.

## Figures and Tables

**Table 1:** Total distribution spectral chart elemental content analysis table.

Element	Line Type	Wt%	Wt% Sigma	At%
O	K Line System	32.94	0.52	59.85
Al	K Line System	13.29	0.15	16.58
Pd	L Line System	0.28	0.11	0.04
Sn	L Line System	53.42	0.75	23.44
Pt	M Line System	0.07	0.27	0.09
Total volume		100.00		100.00

**Table 2:** Information sheet on the instruments used in the SAW sensor gas sensing test system.

Instrument Name	Model	Manufacturers
Frequency counter	SS7200A	Shijiazhuang Digital English Instruments Co, Shijiazhuang, China.
Mass flow meters	D08–4F	Beijing Seven Star Huachuang Co, Beijing, China.
Mass flow controllers (MFCs)	D07–19B	Beijing Seven Star Huachuang Co, Beijing, China.
DC regulated power supplies	WYK–303	Huatai Power Supply Co, Henan, China.
Differential frequency test modules	SDL2101	Beijing Zhongxun Sifang Technology Co, Beijing, China.
Two-way valves	K06–06	Beijing Zhongxun Sifang Technology Co, Beijing, China.
Gas storage cylinders	–	Shanghai Shenyuan Scientific Instruments Co, Shanghai, China.

## Data Availability

The data presented in this study are available on reasonable request from the corresponding author.
